# Supplementary material for: Analysis of Genes Associated with Feeding Preference and Detoxification in Various Developmental Stages of Aglais urticae
Source: Insects. 2024 Jan 3;15(1):30. doi: 10.3390/insects15010030 (PMC10816842; doi:10.3390/insects15010030)
Supplement: Supplementary file 1 [file insects-15-00030-s001.zip › Table S1 Primer used for qRT-PCR analysis.pdf]

Table S1 Primer used for qRT-PCR analysis

Gene	Primer sequence (5'~3')
beta-actin-F	AATGCTGTGATTTCCTTCTGC
beta-actin-R	CTCTCTTCCAGCCCTCATTCT
CarE-F	GTAGTATCATCAGTAATGGGAAACG
CarE-R	TGCCAAAGGAATATCCAGAAA
GST-F	TCCACAACACACAGTTCCGAC
GST-R	AATCTTTCGTCAACTATGCCTCTC
SOD-F	AAATGTGCGGTTTGTCGG
SOD-R	CACCAGCGTTTCCAGTCG
CAT-F	CCTGGTACTAAATGTGCTGGG
CAT-R	AAATGGAATCCTTTTGACTTGAC
