# Supplementary figures and images for: Analysis of Genes Associated with Feeding Preference and Detoxification in Various Developmental Stages of Aglais urticae
Source: Insects. 2024 Jan 3;15(1):30. doi: 10.3390/insects15010030 (PMC10816842; doi:10.3390/insects15010030)

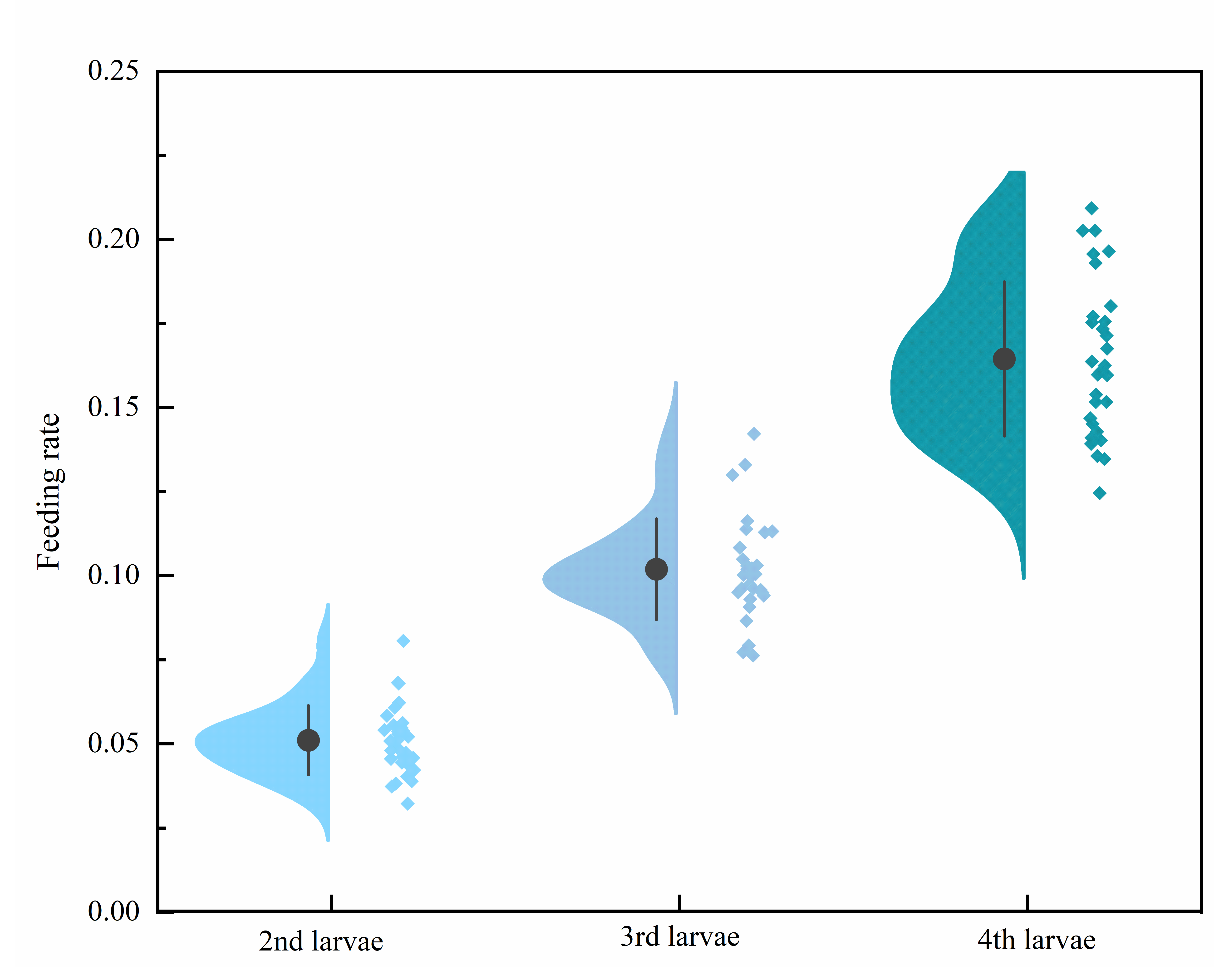

Supplement: Supplementary file 1 [file insects-15-00030-s001.zip › Figure S1 Feeding rate of individual Aglais urticae larvae at different instars.tif]

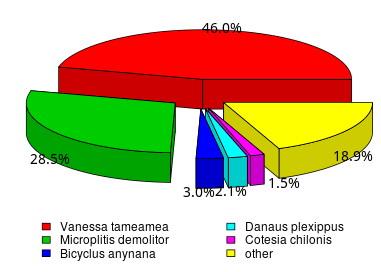

Supplement: Supplementary file 1 [file insects-15-00030-s001.zip › Figure S2 Species distribution of the unigenes annotated in Nr database.png]

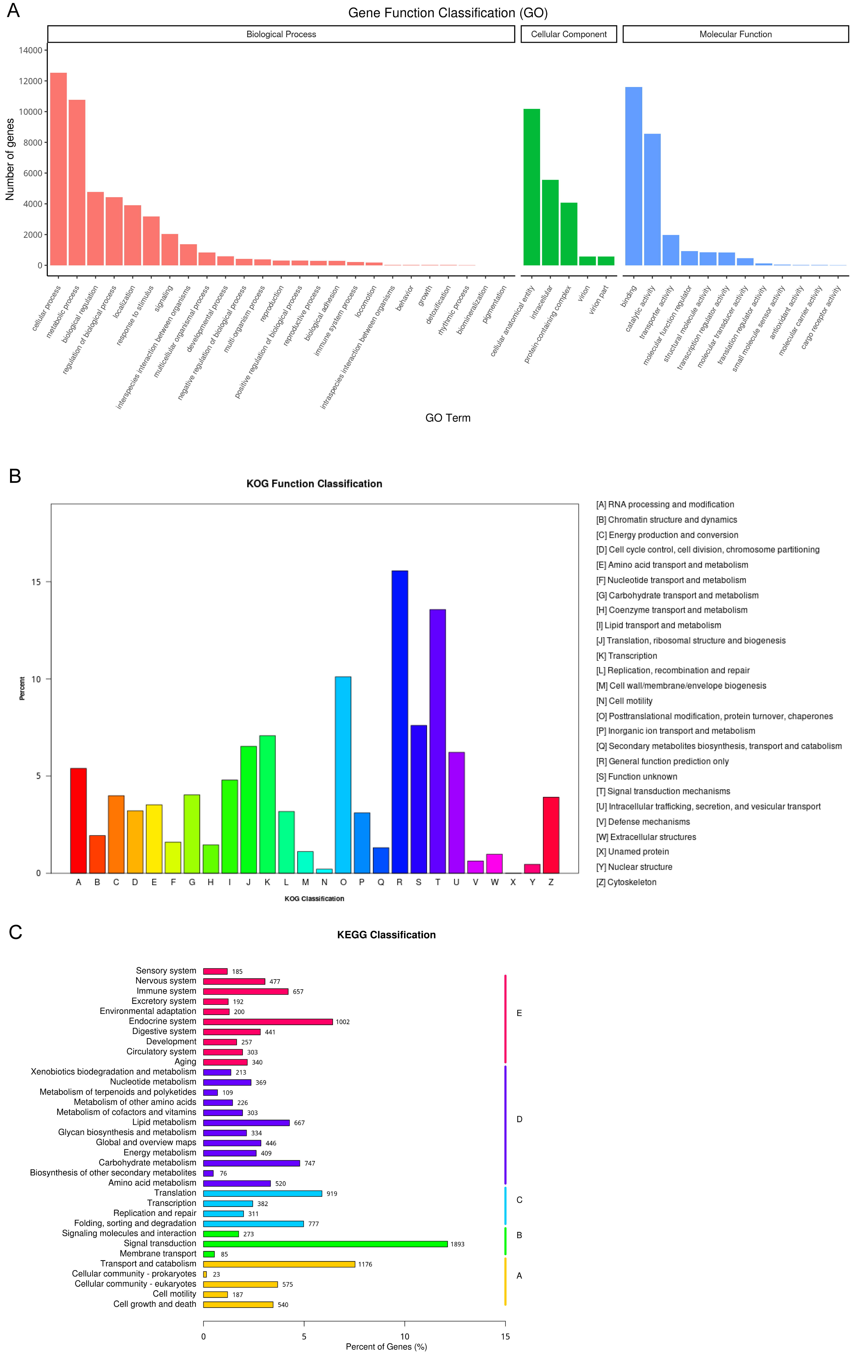

Supplement: Supplementary file 1 [file insects-15-00030-s001.zip › Figure S3 Gene Ontology (GO), Eukaryotic Ortholog Groups (KOG) and Kyoto Encyclopedia of Genes and Genomes (KEGG) classification of transcripts of Aglais urticae.tif]
